# Development of a PET Scanner for Simultaneously Imaging Small Animals with MRI and PET

**DOI:** 10.3390/s140814654

**Published:** 2014-08-12

**Authors:** Christopher J Thompson, Andrew L Goertzen, Jonathan D Thiessen, Daryl Bishop, Greg Stortz, Piotr Kozlowski, Fabrice Retière, Xuezhu Zhang, Vesna Sossi

**Affiliations:** 1 Montreal Neurological Institute, McGill University, 11870 Lavigne, Montreal, QC H4J1X8, Canada; 2 Department of Radiology, University of Manitoba, 807L-715 McDermot Ave. Winnipeg, MB R3E 3P4, Canada; E-Mail: goertzea@cc.umanitoba.ca; 3 Department of Physics and Astronomy, University of Manitoba, Winnipeg, MB R3E 3P4, Canada; E-Mail: xuezhu.zhang@hotmail.com; 4 Imaging Program, Lawson Health Research Institute, 800 Commissioners Road East, London, ON N6A 5W9, Canada; E-Mail: jthiessen@lawsonimaging.ca; 5 Department of Medical Biophysics, University of Western Ontario, Medical Sciences Building Room M407 London, ON N6A 5C1, Canada; 6 Detector Development Group, TRIUMF, 4004 Westbrook Mall, Vancouver, BC V6T 2A3, Canada; E-Mails: daryl@triumf.ca (D.B.); fretiere@triumf.ca (F.R.); 7 Department of Physics and Astronomy, University of British Columbia, 2350 Health Sciences Mall, B3711 Vancouver, BC V6T 1Z3, Canada; E-Mails: gregstortz@gmail.com (G.S.); vesna@phas.ubc.ca (V.S.); 8 Department of Radiology, University of British Columbia, 2350 Health Sciences Mall, B3711 Vancouver, BC V6T 1Z3, Canada; E-Mail: Piotr.Kozlowski@ubc.ca; 9 Biomedical Engineering, University of California, Davis, 451 East Health Sciences Drive Davis, CA 95616, USA

**Keywords:** positron emission tomography (PET), magnetic resonance imaging (MRI), silicon photo-multipliers (SiPMs), block crystal arrays

## Abstract

Recently, positron emission tomography (PET) is playing an increasingly important role in the diagnosis and staging of cancer. Combined PET and X-ray computed tomography (PET-CT) scanners are now the modality of choice in cancer treatment planning. More recently, the combination of PET and magnetic resonance imaging (MRI) is being explored in many sites. Combining PET and MRI has presented many challenges since the photo-multiplier tubes (PMT) in PET do not function in high magnetic fields, and conventional PET detectors distort MRI images. Solid state light sensors like avalanche photo-diodes (APDs) and more recently silicon photo-multipliers (SiPMs) are much less sensitive to magnetic fields thus easing the compatibility issues. This paper presents the results of a group of Canadian scientists who are developing a PET detector ring which fits inside a high field small animal MRI scanner with the goal of providing simultaneous PET and MRI images of small rodents used in pre-clinical medical research. We discuss the evolution of both the crystal blocks (which detect annihilation photons from positron decay) and the SiPM array performance in the last four years which together combine to deliver significant system performance in terms of speed, energy and timing resolution.

## 1. Introduction

### 1.1. Positron Emission Tomography (PET) Basics

Positron Emission Tomography (PET) exploits the fact that many biologically significant molecules can be labelled with a positron emitting isotope of carbon or fluorine which can be imaged to show the distribution of that molecule or its metabolites in humans and animals. ^11^C and ^18^F decay by positron emission and the positron, after losing its initial kinetic energy, annihilates with an electron producing two 511 keV photons which are emitted approximately 180° apart. These annihilation photons travel beyond the body and are detected by arrays of dense, optically transparent scintillation crystals which convert some of that photon's energy into visible light. This light is normally converted into an electrical pulse by a photo-multiplier tubes (PMT). PET scanners' detectors normally have an array of crystals optically coupled to four PMTs. Signals from pairs of detectors which arrive almost simultaneously identify the line on which the positron decay occurred, and many such detections are submitted to a reconstruction algorithm which is used to produce a map of the density of positron decays from which quantitative maps of the labelled molecules' distribution can be derived as a function of time. The interested reader is referred to three texts dealing with clinical PET [1], brain imaging with PET [2], and reconstruction in modern PET scanners [3]. Most PET scanners have their detectors arranged in cylinders with considerable axial extent. This allows for a very large number of lines of response since all chords, (including those oblique to the scanner axis) which intersect the subject being imaged are used in the image reconstruction.

During the signal acquisition, several effects will occur that lead to incorrect information about the location of the decay: (1) the positron escapes from the parent nucleus with a variable energy which must be lost before annihilation with an electron can occur so there is a small difference between the location of the nucleus and annihilation (positron range error); (2) the finite size of the crystals which detect the annihilation photons adds a further uncertainty to the precise location of the line of response for this event (crystal size error); (3) the electron-positron pair will be moving at the time of annihilation, so the two annihilation photons will not appear to be quite 180° to the stationary detectors (non-colinearity error); (4) one or more of the photons may undergo Compton scattering within the body prior to detection. In clinical PET the errors associated with the first two of these effects are normally small compared to the errors associated with subject movement during the scan or mis-registration of the PET and computed tomography (CT) images which are acquired at different times. However, when imaging small animals the effects of positron range and detector size are much more significant while non-colinearity produces much smaller errors due to the smaller scanner size [4]. Compton scattering also occurs in the detectors, and this results in a broadening of the spatial resolution, since the detector may assign the point of interaction to than of the centroid of all the interactions within the detector.

### 1.2. Small Animal PET Requirements

Compared with whole body PET scanners, small animal PET scanners require a much smaller field of view. Due to the smaller organ size, much higher spatial resolution is required. For more detailed information, the reader is referred to a basic text on small animal imaging [5]. With a smaller scanner diameter, the effect of non-colinearity is much reduced. Since there are fewer detectors, given the overall size of the scanner is much smaller, the scintillation crystals can be made smaller while still keeping the device cost effective. Since the crystal size is a major contributor to blurring, this is very important. The physical size of conventional PMTs makes them unsuitable for coupling to the very small crystals used in small animal PET. Therefore most of them use position sensitive PMTs (PSPMTs). These use mesh dynodes rather than those in a conventional PMT which are usually arcs of a cylinder. They also have two orthogonal sets of parallel wires as anodes, so that the spatial distribution of charge can be determined. Since the dynodes are a stack of meshes the distribution of charge collected at the anodes reflects quite faithfully the location of the light detected by the photo-cathode. An excellent review of multi-modal small animal imaging has been published by de Kemp *et al.* [6].

### 1.3. University of Sherbrooke PET Scanners

In 1990, the group at the University of Sherbrooke made the first small animal PET scanner in Canada [7,8]. For many years, it was unique in that it used a solid state light sensor, an avalanche photo-diode (APD) rather than a PMT. These are much smaller than the smallest practical PMT, require a much lower operating bias (∼100 V *vs.* 1000 V for a PMT), and are not adversely affected by magnetic fields. This instrument demonstrated many of the advantages of small animal PET imaging compared to highly invasive methods like autoradiography, the most significant of which are that an animal can be used as its own control, and that dynamic imaging of the radioisotope distribution is possible. Later a much more advanced version of their LabPET was commercialized [9] and has been very successful.

### 1.4. Scintillators for PET

The annihilation photons imaged by PET scanners have a much higher energy, 511 keV, than the 140 keV gamma rays from ^99m^Tc used in conventional nuclear medicine imaging. Since it is very important to absorb all the energy from the incident photon (in order to measure its energy and distinguish between true and scattered photons), a scintillator with both a high atomic number (to favor photo-electric detection) and high density (to improve overall efficiency), PET scanners use high atomic number, high density scintillators. In the 1980s and early 1990s Bismuth Germanate (BGO), (first described in 1975 [10]), was the scintillator of choice due to its high effective atomic number, and very high density. In fact, the first PET scanner to use BGO was made in Canada in 1978 [11], and this scintillator was adopted by nearly all commercial PET manufacturers subsequently. More recently, rare earth based scintillators have been shown to be much more efficient in converting the incident photon energy into visible light, and also their decay time is much shorter, resulting in lower dead time, and allowing higher count rates. The first of these, gadolinium oxy-orthosilicate (GSO) was used in some scanners. Lutetium oxy- orthosilicates doped with cerium have been the most promising [12], at present, the materials of choice are lutetium oxy-orthosilicate (LSO) or lutetium-yttrium oxy-orthosilicate (LYSO), in which a small quantity of yttrium is added to change the decay time constant and avoid patent infringement. The properties of some commonly used scintillators are given in Table 1.

### 1.5. Block Detectors: Encoding of Data

The early PET scanners had one scintillation crystal coupled to one PMT, but this is very expensive and prevents making the detectors sufficiently small to improve the spatial resolution. Almost all PET scanners now use some form of “block detector” [13] in which many crystals are optically coupled to four PMTs. Small animal PET scanners use position-sensitive PMTs (PS-PMTs) rather than individual PMTs which allow them to identify the small crystals in the block more easily. As an example, the Siemens Biograph family of PET/CT scanners' including the Biograph 16 HiRez [14], use detector blocks that have 169 crystals (each 4 × 4 × 20 mm^3^) arranged in a square array coupled to four PMTs. In comparison, the Siemens Inveon small animal PET scanner [15] has detectors with 400 crystals (each 1.5 × 1.5 × 10 mm^3^) on a single PS-PMT.

**Figure 1.**
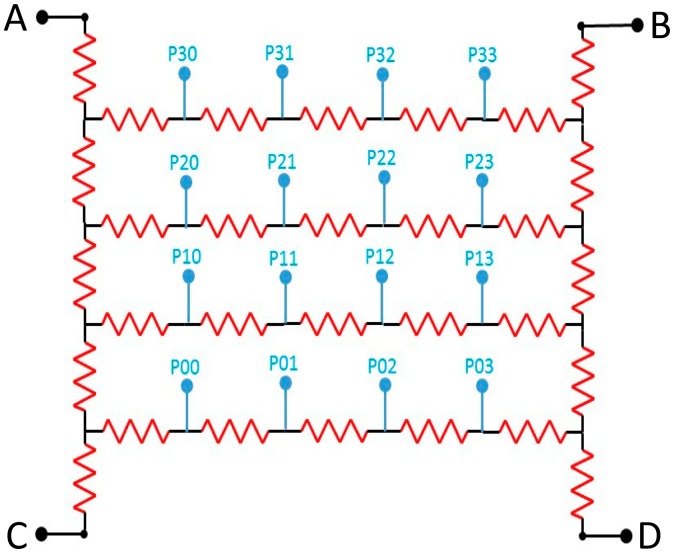
Simplified circuit of the 16:4 resistor network used to reduce the number of outputs from a 4 × 4 array of SiPMs to four outputs labeled *A*, *B*, *C* and *D*.

The ability to identify uniquely each of the crystals in the block is critical to the spatial resolution of the final instrument. Since LSO has a much higher light output than BGO, the regions associated with each crystal in the crystal identification map (CIM) are much smaller due to the higher signal to noise ratio (SNR). The map is formed using what is often referred to as “Anger Logic” in the PET literature in honor of Hal Anger, inventor of the Gamma Camera [16]. The four outputs from the PMTs or ends of a series of resistors connecting the anodes of a PSPMT as shown in Figure 1, can be considered in a pattern like:

$$\begin{array}{cc} \text{A} & \text{B}\\ \text{C} & \text{D} \end{array}$$

From total light collected, corresponding to the energy, *E*, and the individual output signals *A*, *B*, *C*, and *D* the coordinates, *X*, *Y* of that interaction can be calculated according to:

(1) E=(A+B+C+D),X=B+D/E,Y=(A+B)/E

**Figure 2.**
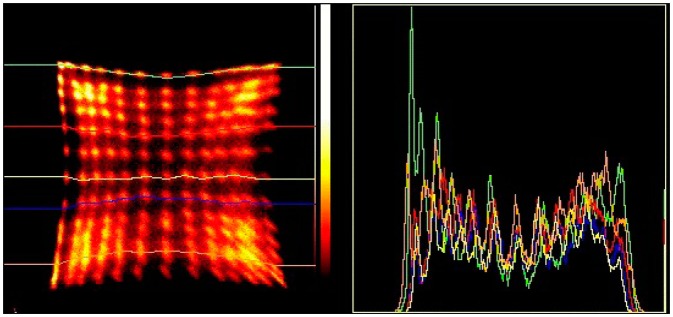
Crystal identification map from a Siemens HiRez 169 LSO crystal PET detector.

**Figure 3.**
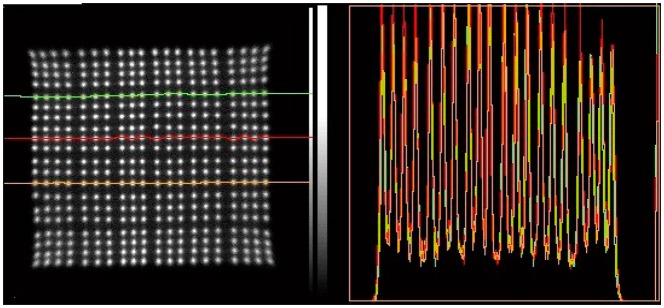
Crystal identification map from an Inveon 400 LSO crystal pre-clinical PET detector.

The above equations were used to make the CIM images in Figures 2 and 3.

### 1.6. SiPMs

Silicon photomultipliers [17] have become available in recent years and their properties are being exploited by many groups for PET imaging [18]. These devices are made from a large number of miniature Geiger-mode APDs, (commonly referred to as cells) all connected in parallel. SiPMs are operated by biasing them just beyond their breakdown voltage, so that each cell fires in an avalanche (*i.e.*, Geiger) discharge in response to incoming light photons. The proportionality of the signal thus arises through integrating or counting the number of cells that have fired. Thus, the more cells receiving light, the bigger the signal detected. Arrays of SiPMs, in sizes of 4 × 4 or larger, are now available for PET applications from at least three manufacturers: Hamamatsu, Japan [19], Philips, Germany [20] and SensL, Ireland [21].

### 1.7. Digital vs. Analog SiPMs

**Figure 4.**
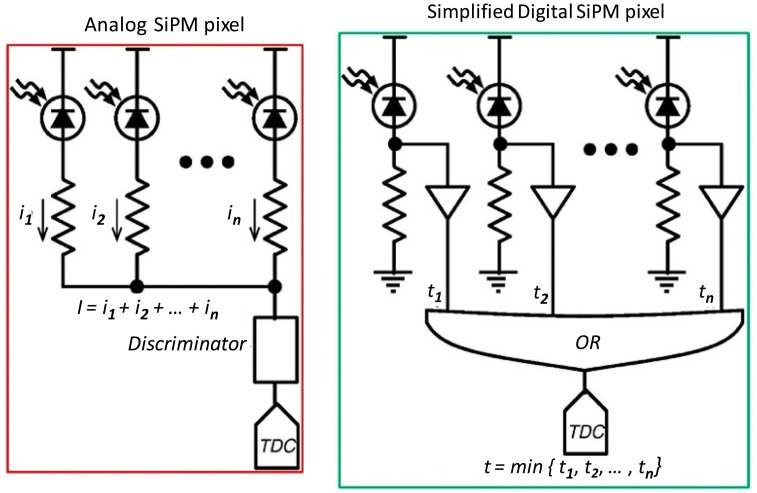
(**a**) Analog and (**b**) digital SiPM cells showing how an event trigger is formed in both configurations. Reproduced with the author's permission from IEEE MIC conference record 2012 [23].

The SiPMs made by Hamamatsu and SensL are formed from a few thousand light sensitive APDs connected via resistors to a common point. These APDs are referred to as “cells”. The concept is illustrated in Figure 4a. The sum of all the APD cells' outputs are connected to a common point whose output is sent to a discriminator which produces a trigger. Some of the cells will fire spontaneously, and the higher the bias, the more cells will fire in a given time. This constitutes a background noise. On the other hand, the digital photon counters (DPCs), from Philips are very versatile. Their simplest mode of operation has the cells conceptually coupled to form a trigger through a logic “OR” gate, as shown in Figure 4b, and once a trigger has occurred, the state of all the APDs is interrogated a short time later, to count the number which fired. In this way, the time of the first cell to trigger is a very precise measure of the actual time the scintillator detected an incoming photon. The analog SiPMs are used in circuits very similar to those used with conventional PMTs, except that the bias voltage is very much lower. DPCs have a very sophisticated integrated circuit attached, but still require additional logic to validate and encode events. Application to PET is described in a recent article by our group [22].

Most of the research to date by our group has been based on the tiled arrays made by SensL Inc. of Cork, Ireland. The work described here summarizes the evolution of our detector design for use in a prototype small animal MRI compatible PET scanner which is presently under construction.

### 1.8. SensL SiPMs: Families

#### 1.8.1. Single and Tiled Arrays

Over the past five years, SensL has offered 4 × 4 arrays of SiPMs in a package which can be “tiled” in order to make larger arrays. These are referred to by SensL as: SPMArray 4, Array SL-4, Array SM-4, and Array SB-4. The first version we used could be tiled (*i.e.*, close packed) on three of the four sides and a thin, flat and flexible cable extended from the remaining side. The more recent versions have the read-out pins on the back, and can be tiled on all four sides.

#### 1.8.2. Evolution of Performance

Each of the elements is 3.05 mm × 3.05 mm with a pitch of 3.16 mm. Their parameters are given in Table 2. In general the gain has improved by a factor of three, and is now comparable to a conventional PMT. The latest versions need only a bias voltage of about 24 V.

## 2. Materials and Methods

### 2.1. Block Detectors Used in Our Evaluation

**Figure 5.**
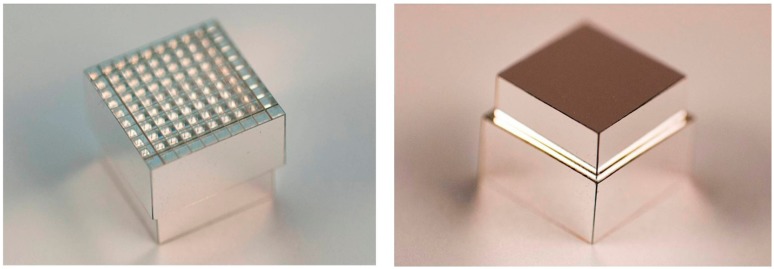
Two views of block of LYSO crystals used in the study. There are 10 × 10 1.2 mm crystals in the lower layer (shown exposed here) and 9 × 9 1.2 mm crystals in the upper layer. The layers are offset by ½ of the crystal spacing.

As mentioned above, most PET scanners use a “block detector” designed so that a larger number of individual crystals can be coupled to a smaller number of light sensors. The design of these has evolved over the years and the choice is critical in optimizing the instruments spatial resolution and overall cost to the task of human whole-body, human brain, or small animal imaging. Our current PET detector is designed for a Bruker 7T MRI with a 21 cm horizontal bore and a Bruker BGA12-S shielded gradient system with an inner/outer diameter of 114 mm/198 mm (Bruker BioSpin, Milton, ON, Canada) [24]. One of the constraints in our instrument is that the PET detector must fit inside the gradient coils while also accommodating a radio frequency coil. Currently, we are using a quadrature volume RF coil with inside and outside diameters of 35 and 60 mm (Bruker BioSpin, Milton, ON, Canada), thus limiting the inner diameter of the detector ring to approximately 64 mm. Conventional PET detector crystals are much longer in the radial direction than in their other dimensions. This results in the PET image becoming blurred in the radial direction due to the uncertainty of the point of interaction of the incoming photon. For this project we mitigated this effect using a dual layer crystal block [25]. These blocks have two layers with the upper one shorter than the lower layer to make the probability of interaction the same in both layers. In order to distinguish the layer of interaction, we make the upper layer with one fewer rows and columns and offset it by ½ of the crystal pitch. Our first experiments used arrays made in-house by hand with individual crystals in a 4 × 4 and 3 × 3 pattern [26], while later ones were commercially manufactured by Proteus, Inc. (Chagrin Falls, OH, USA) [27], allowing the use of much finer crystal elements. The use of offset crystal elements was originally developed for the first successful dedicated PET scanner for breast cancer detection [28,29]. The crystal blocks fabricated by Proteus for this project have properties as shown in Table 3. Figure 5 shows the exposed 10 × 10 lower layer and the outer surface of the enclosed 9 × 9 upper layer of the LYSO block whose performance is described here. The crystals are 1.2 × 1.2 mm^2^ and 6 mm deep in the lower layer and 4 mm deep on the upper layer.

The crystal blocks are designed to fit on the SensL 4 × 4 arrays. The number of crystals one can uniquely identify determines the packing density. Using sensors which have less noise makes the blobs associated with each crystal smaller, allowing one to encode more crystals. In this regard, position sensitive PMTs presently provide the best performance as can be seen comparing the response patterns in Figures 2 and 3. Improvements in the photo-detection efficiency and dark current have helped SiPMs come very close to matching their performance. The use of 3M “enhanced specular reflector” (ESR) [30] in the fabrication of the crystal blocks improves the light output significantly over aluminum foil or Toray reflector [31].

### 2.2. Key Factors in Performance

#### 2.2.1. Crystal Detectability

The ability to uniquely identify the crystal of interaction of the incoming photon is critical to the ultimate spatial resolution of the PET scanner. This is already compromised by the physics of the interaction of gamma rays with matter which favors Compton scattering (where only part of the incoming photon's energy is lost in the material in the first interaction) over photo-electric absorption (where all the energy is lost at one point in the material). High atomic number materials favor photo-electric absorption, but for scintillation crystals used in PET this is never more than 40%, and is only 30% for LSO, and even less for LYSO, depending of the percentage of yttrium used in the crystal. The detectability is improved with higher light output, better reflectors between crystals, and lower noise light sensors, all of which serve to enhance the SNR. Our use of a ½ crystal offset in both directions [28] allows for more crystals to be identified since the light from the top layer appears to come from a point in the center of a square formed by the four crystals below the top layer crystal.

#### 2.2.2. Energy Resolution

Energy resolution is important in PET imaging as it allows the scanner to distinguish between annihilation photons which have been Compton scattered in the subject or intervening material and those which are still on their original line of response. The energy resolution is enhanced by higher light output, better reflectors between crystals, and lower noise light sensors.

#### 2.2.3. Timing Resolution

Timing resolution is important in PET as it allows one to distinguish between the detection of two annihilation photons from the same event, and two which arise from two different positron-electron annihilations which occur closely separated in time. In some of the newer clinical PET scanners the arrival time difference is used to estimate the approximate point along the line of response where the annihilation occurred, so called time-of-flight PET. However current PET detectors are not fast enough to make this practical in small animal imaging.

### 2.3. Readout: Use of Multiplexing and HDMI Cables

The SensL devices we use have 16 elements arranged in a 4 × 4 matrix. For simplicity these are multiplexed to provide only four connections. An array of resistors couples the outputs together so that they can be encoded in the same manner as a conventional PET detector shown in Section 1.5. The signals are processed on a transmitter board which has a socket to receive the SensL SiPM array at one end and a miniature high definition media interface (HDMI) connector at the other. The HDMI cables have four twinax 50 ohm cables which normally carry the video signals to a television receiver [32,33]. These are re-assigned to carry the four signal outputs, while other conductors provide the + and −3 V power for the multiplexer, and ∼25 V bias for the SiPMs. A thermometer chip monitors the temperature. These cables transmit the signals from the PET detectors very faithfully even in the presence of the MRI imaging sequences which generate the highest RF noise due to the differential signals in the shielded twinax cables. No magnetic components are used in the manufacture of the SiPMs or the multiplexer, so the introduction of the PET detectors does not influence the MRI acquisition.

**Figure 6.**
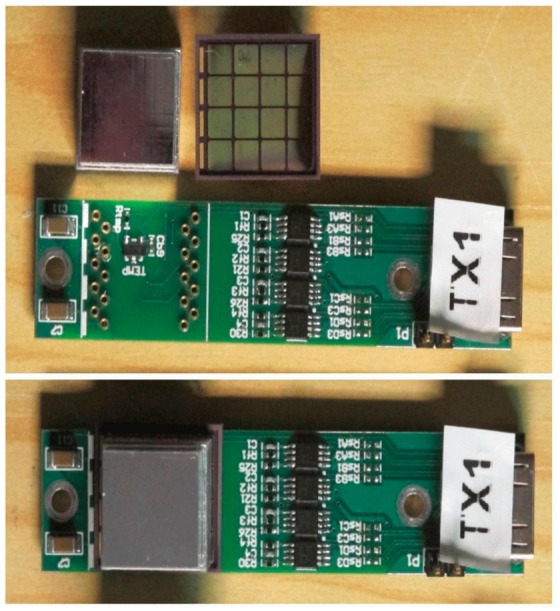
Detector assembly consisting of the multiplexer, SiPM array and crystal block.

The multiplexer is shown in Figure 6. The two views show the detector fully assembled, and the multiplexer circuit board with its miniature HDMI connector at one end and the crystal block mounted on the SiPM array at the other end.

### 2.4. Experiments to Validate the Detector Performance

#### 2.4.1. Energy and Timing Resolution

The single detector crystal identification, energy and timing resolution were performed using a Scanwell PET timing alignment probe [34] containing a ^22^Na source as described [35]. Six channels of list-mode data were acquired (four detector outputs and two time to amplitude converter (TAC) outputs. The list mode file was played back to form a crystal map on which the response of all crystals was identified. Then the list file was replayed with the four channels of data from each crystal forming an amplitude histogram from which the location of the 511 keV peak was found and the energy resolution was measured in terms of the full width at half maximum (FWHM) of a Gaussian fit to the photo-peak. Subsequently the list file was replayed and the time between the positron decay being detected in the timing probe and the subsequent detection by individual crystals was formed into a timing spectrum and the time delay and timing resolution's FWHM measured.

#### 2.4.2. Spatial Resolution

In these studies two identical detectors were attached to large protractors supported on translation stages. A stationary ^22^Na point source was placed centrally between the detectors and data were collected with the two detectors operating in coincidence. The detectors were rotated to positions corresponding to the locations of the ends of chords which intersect the centers of the detectors as if there were 16 detectors in a tightly packed “ring”. The detectors were moved in 0.2 mm increments making 60 measurements of the count-rate in each position. The data from each column of crystals were summed to measure the coincidence response function (CRF) as a function of position in the block and radial offset of the lines of response joining the crystal columns. The FWHM of the CRF was then plotted as a function of the distance from the center of the scanner's field of view.

## 3. Results

### 3.1. Crystal Identification Maps

**Figure 7.**
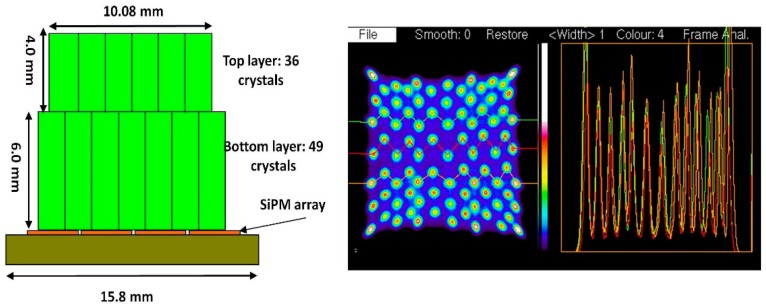
Detector with SensL S-series SiPM and LYSO crystal block with 36 crystals in the top layer and 49 in the bottom layer and the response pattern with profiles through rows of crystals showing that they are well resolved.

**Figure 8.**
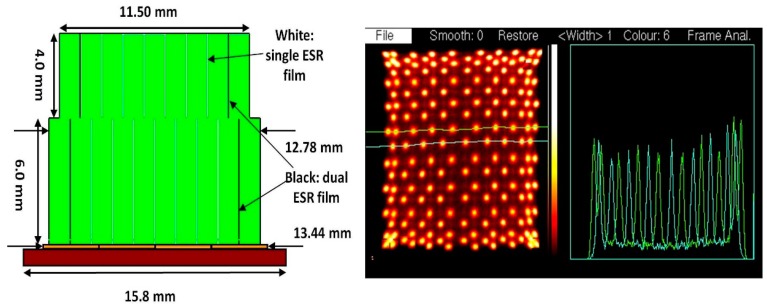
Detector with SensL B-series SiPM and LYSO crystal block with 81 crystals in the upper layer and 100 in the lower layer and the response pattern with profiles through rows of crystals showing that they are well resolved.

The crystal identification maps are used to assign one part of the image formed by data processed according to Equation 1 to a unique crystal. We present the results of 7 × 7 + 6 × 6 arrays of 1.6 mm crystals mounted on the older SensL L-series SiPMs in Figure 7, and 10 × 10 + 9 × 9, arrays of 1.2 mm crystals mounted on the newer SensL B-series SiPMs in Figure 8. These figures show the crystal arrangement and sizes and profiles through the individual crystals' responses. There are 84 crystals in the older block, and 181 in the newer one. Due to a large extent on the lower noise in the SiPMs all crystals appear just as well resolved in the newer version.

### 3.2. Energy resolution

**Figure 9.**
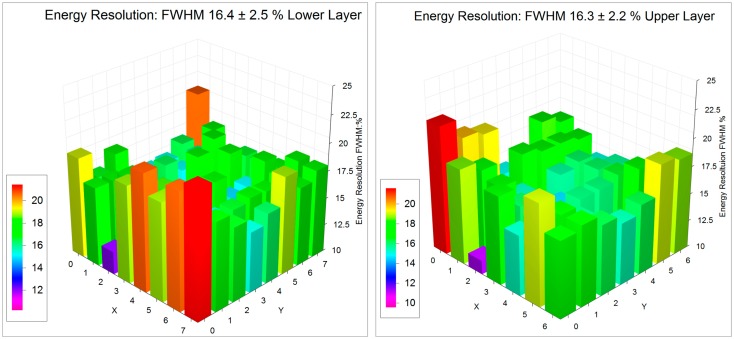
Energy resolution expressed at the FWHM % for the 511 keV photo-peak for the lower and upper layer of the 8 × 8 lower, 7 × 7 upper layer block on a SensL M-series SiPM.

**Figure 10.**
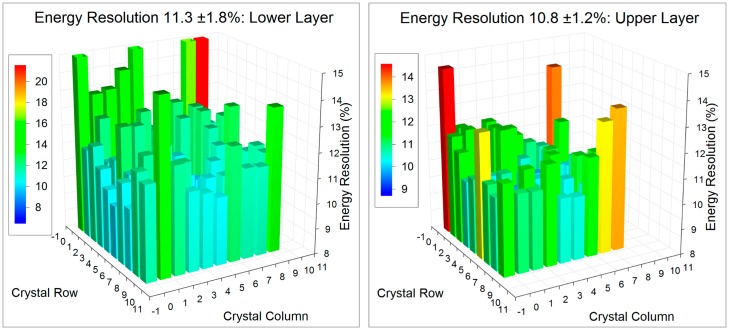
Energy resolution expressed at the FWHM % for the 511 keV photo-peak for the lower and upper layer of the 10 × 10 lower, 9 × 9 upper layer block on a SensL B-series SiPM.

The energy resolution and the ability to distinguish between direct and scattered incoming photons is important for improving the signal to noise ratio and the contrast in the final PET images. By way of example we present 3D histograms from individual crystals of the energy resolution and location of the 511 photo-peak for 8 × 8 + 7 × 7 1.2 mm crystals mounted on the SensL L-series SiPMs in Figure 9, and similar figures for the 10 × 10 + 9 × 9 1.2 mm crystals on the newer SensL B-series SiPMs in Figure 10. The energy resolution improves from 16.4% to 11.3% FWHM in the lower layer and from 16.3% to 10.8% in the upper layer of crystals even though there are 23% more crystals in the array.

### 3.3. Timing Resolution

**Figure 11.**
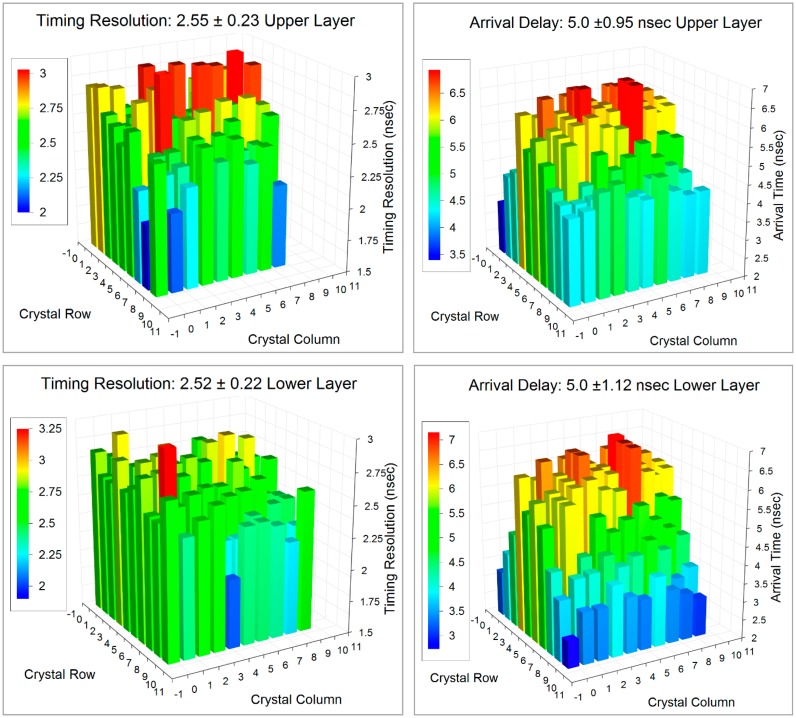
The timing resolution and arrival time delays for the dual layer block plotted as a function of crystal location in the block.

Improved timing resolution reduces the statistical noise in the final PET. The timing resolution of the 10 × 10 + 9 × 9 1.2 mm crystal blocks on the SensL SB-series SiPMs is shown in Figure 11 for the lower layer: as 2.52 ± 0.22 ns FWHM and upper layer as 2.55 ± 0.22 ns FWHM. The crystal to crystal variation in timing resolution appears quite random. However, the arrival time delays (which are normalized to 5 ns) show a trend with the events detected near the center of the block appearing to arrive later. This is due to the nature of the multiplexer used in which the 16 individual SiPMs are connected with a resistor matrix to provide only four channels to read out. The inter-element resistors and the intrinsic capacitance of the SiPMs themselves delays the arrival of the signals: the further away the element is from the corner, the longer it takes for the signal to arrive. These delays can easily be accounted for in the final instrument at the time the system is calibrated.

### 3.4. Spatial Resolution

**Figure 12.**
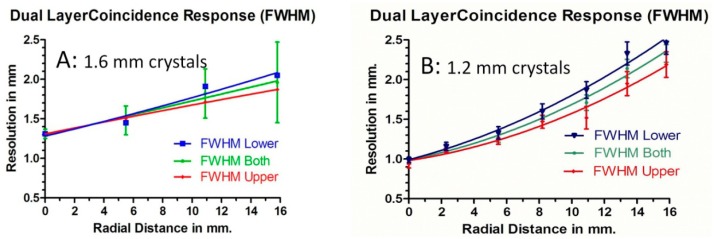
The coincidence response function as it changes with radial distance for (**A**): a block with 49 lower, and 36 upper layer crystals 1.6 × 1.6 mm^2^; (**B**): 64 lower and 49 upper layer crystals 1.2 × 1.2 mm^2^.

**Figure 13.**
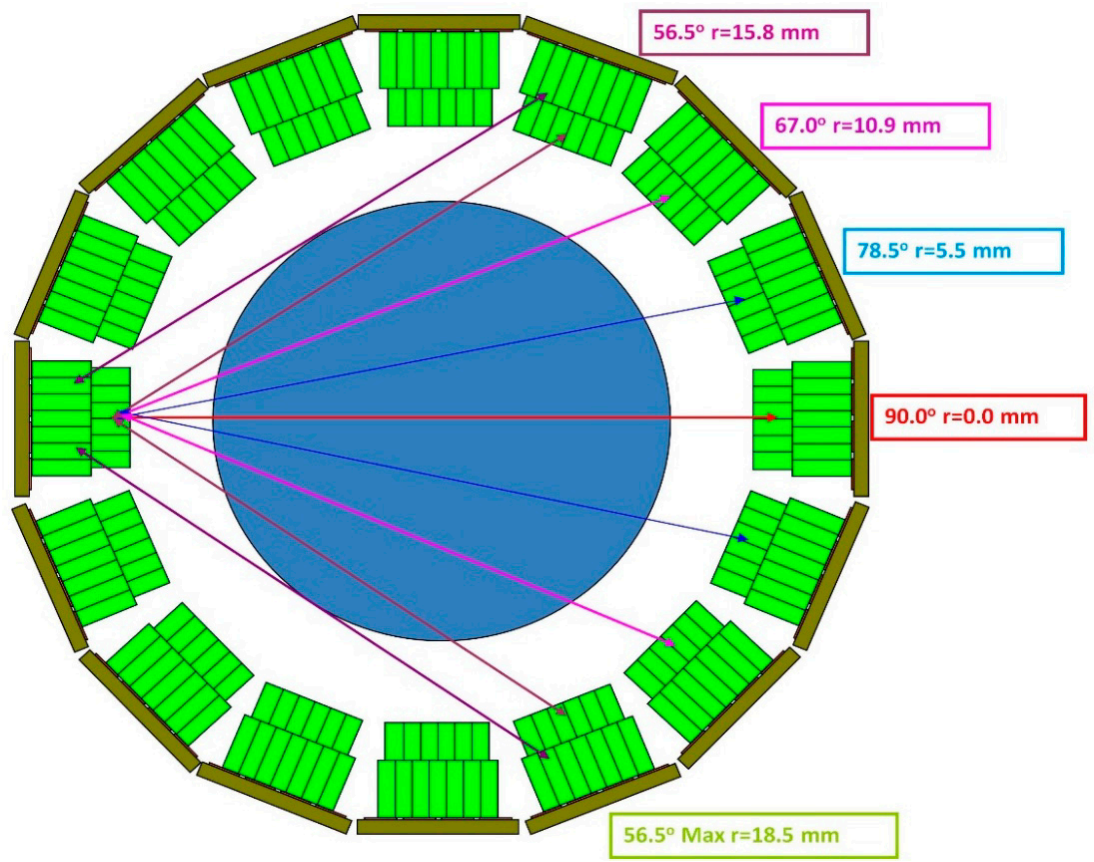
Trans-axial view of the detectors and a disk covering the imaging field of view. The colored chords show lines of response which intersect the crystals more obliquely as they are further from diameter.

The spatial resolution of the final PET scanner depends on the coincidence response function of the detector pairs to a point source as a function of the location in the imaging field and the reconstruction algorithm. We present the results of the coincidence response function for two different crystal blocks: dual layer 7 × 7 + 6 × 6 1.6 mm crystals and 8 × 8 + 7 × 7 1.2 mm crystals in Figure 12. Figure 13 shows the detector ring geometry in one plane to illustrate where those measurements of spatial resolution are made as chords which are at different radii.

## 4. Conclusions/Outlook

The funding for the construction of a dual-ring demonstration prototype has been secured, and construction of the instrument is presently underway. Extensive analytical and Monte Carlo simulations [36,37] were performed prior to the final design, and these agree well with the spatial resolution results reported here. The instrument will have two rings of 16 SiPMs on which we will mount LYSO blocks of 22 × 10 1.2 mm crystals on the lower layer, and 21 × 9 1.2 mm crystals on the upper layer: (409 crystals altogether). The detectors will have individual thin copper shielded boxes to minimize the RF pickup during MRI acquisitions. Each block will be readout with a single HDMI cable. A controller [38] will monitor the temperature of each crystal and adjust its bias slightly as the temperature changes which will occur when MRI pulse sequences requiring rapidly changing gradients are being used. The readout of the detectors will use the OpenPET data acquisition system which has been developed for groups constructing small PET systems by the Lawrence Berkeley National Laboratory group [39]. Many other are actively working in this field at present for example Maramraju *et al.* [40] used a higher field (9.4 T) magnet and unshielded PET detectors. They experienced some interference between modalities, which so far, with our shielded PET detectors and HDMI cables has not been observed in our prototype. Already there is a commercial MRI/PET scanner using a low field (1 T) permanent magnet offered for sale by Mediso USA [41]. Dual modality MRI and PET present some interesting design challenges, but our preliminary results presented here and the MRI compatibility results to be published shortly suggest that we have a solution which should provide excellent bi-modal imaging at a reasonable cost.

## Figures and Tables

**Table 1.** Common scintillators use in PET imaging, compiled from various sources.

**Properties**	**GSO**	**BGO**	**LSO**
Density (g/cc)	6.71	7.13	7.35
Effective Atomic Number	58	73	65
Index of Refraction	1.87	2.15	1.82
Attenuation (cm^−1^ @ 511 keV)	0.67	0.96	0.87
Decay Constant (ns)	50	300	40
Light Yield Nal (T1) = 100 (PMT, APD)	20, 40	15, 30	75, 85
Photoelectric-fraction (% @ 511 keV)	25	40	30
Energy Resolution (% @ 511 keV)	15	20	12
Radioactivity	None	None	^176^Lu

**Table 2.** Specifications of SensL 4 × 4 SiPM arrays used in this work.

**Parameter**	**SPM-Array 2**	**S-Series**	**M-Series**	**B-Series**
Bias voltage	−30 V	+28 V	+28 V	−24 V
Gain	1 × 10^6^	1 × 10^6^	2.3 × 10^6^	3.0 × 10^6^
Area	2.85 × 2.85	2.85 × 2.85	3.05 × 3.05	3.0 × 3.0
Photon Detection Efficiency	10%–20% @ 5200 Å	10%–20% @ 5200 Å	20% @ 5200 Å	31% @ 4200 Å
Number of cells	3640	3640	4774	4774
Dark rate or current	8 MHz	8 MHz	3.8 μA	2.8 μA

**Table 3.** Evolution of dimensions and crystal configurations used.

**Reference**	**Crystal Size**	**Top Layer**	**Bottom Layer**	**Reflector**	**Manufacturer**
26	3 mm × 3 mm	3 × 3	4 × 4	Toray film	hand made
25	1.6 mm × 1.6 mm	6 × 6	7 × 7	ESR	Proteus
25	1.2 mm × 1.2 mm	7 × 7	8 × 8	ESR	Proteus
35	1.2 mm × 1.2 mm	9 × 9	10 × 10	ESR	Proteus
in progress	1.2 mm × 1.2 mm	9 × 21	10 × 22	ESR	Proteus
